# Changes in protein thiols in response to salt stress in embryogenic suspension cultures of *Dactylis glomerata* L.

**DOI:** 10.1080/13102818.2014.946798

**Published:** 2014-10-22

**Authors:** Lyuben Zagorchev, Miroslava Terzieva, Marina Stoichkova, Mariela Odjakova

**Affiliations:** ^a^ Department of Biochemistry, Faculty of Biology, Sofia University St. Kliment Ohridski, Sofia, Bulgaria

**Keywords:** glutathionylation, glutathione, reactive oxygen species, somatic embryogenesis, stress tolerance, thioredoxin

## Abstract

The aim of the present study is to assess the rate of protein disulphide formation and the activity of NADPH-dependent thioredoxin and glutaredoxin systems, responsible for the reverse reduction of protein and mixed protein-glutathione disulphides, in embryogenic suspension cultures of *Dactylis glomerata*, subjected to salt stress. Two concentrations of NaCl previously established as enhancing (0.085 mol/L) and inhibiting (0.17 mol/L) somatic embryogenesis were used. The quantitative (by colour reaction with Ellman's reagent) and qualitative (by diagonal gel electrophoresis) analyses showed a significant increase in protein disulphide formation in salt-treated cultures compared to controls. The ratio of disulphides to free thiols is higher in 0.17 mol/L NaCl-treated cultures. The activity of the thioredoxin–thioredoxin reductase system has been increased accordingly in 0.085 mol/L NaCl-treated cultures but decreased at the higher salt concentration. The activity of glutaredoxins was also estimated, by using glutathionylated bovine serum albumin as substrate and following the decrease of NADPH absorbance at 340 nm in the presence of glutathione and glutathione reductase. Mild salt (0.085 mol/L NaCl) treated cultures again showed the highest activity compared to controls and 0.17 mol/L NaCl-treated cultures. Based on these observations it was suggested that salt treatment resulted in increased protein disulphide formation and thioredoxin and glutaredoxin systems are important regulators of this process, strongly involved in salt stress response. The highest activity at 0.085 mol/L NaCl may be also related to the regulatory mechanisms, involved in the potentiating of somatic embryogenesis at this salt concentration.

## Introduction

Plants are constantly subjected to the changing environmental conditions that eventually lead to stress. Among the abiotic stresses high soil salinity, caused by excessive concentration of NaCl, is a major crop yield reducing factor.[1] Besides the initial osmotic stress and the direct toxicity of Na^+^, salt stress also leads to significant production of reactive oxygen species (ROS).[2] When the antioxidant systems are not able to compensate the overproduction of ROS (condition of oxidative stress), this could lead to oxidative damage of membrane lipids, nucleic acids and proteins. The Cys (cysteine) residues in intracellular proteins are extremely vulnerable to oxidative modifications. Their thiol groups could undergo three stages of oxidation, primary the reversible stage to sulphenic acid (Cys–SOH), then irreversibly to sulphinic (Cys–SO_2_H) and sulphonic (Cys–SO_3_H) acid. The irreversibility of the second stage is however questionable.[3] Proteins with oxidized thiols lose their native conformation and activity and are subjected to degradation.

The reversible formation of intramolecular disulphide bonds and mixed protein-glutathione disulphides are the two major protective mechanisms that cope with oxidative damage of proteins.[4] In the first case, the reverse reduction of disulphide bonds is mediated by interchange of the bond to Cys containing thioredoxins which in turn are reduced by reduced nicotine amide adenine dinucleotide phosphate (NADPH)-dependent flavin adenine dinucleotide (FAD) containing thioredoxin reductases. Intramolecular disulphide bond formation is also a regulatory mechanism that could alter enzymatic activities like the induction of phytochelatin synthase, etc. Protein glutathionylation is a similar mechanism in which a mixed protein-GSH disulphide is formed in a reaction involving Cys-SOH and glutathione disulphide (GSSG). Another class of low molecular weight Cys containing proteins, the family of glutaredoxins (Grx), is involved in the reversible reduction of mixed disulphides. This would lead to formation of GSSG that is reduced to glutathione (GSH) by NADPH-dependent glutathione reductases. Protein glutathionylation is also a regulatory mechanism that could lead to increased or decreased activity of different enzymes.[5]

The aim of the present study is to evaluate the protein thiol and mixed protein-GSH disulphides formation and the activity of the thioredoxin and glutaredoxin systems in salt-treated suspension cultures of *Dactylis glomerata* L. Besides the stress response function, the possible relation and regulatory functions of the mechanisms mentioned above to somatic embryogenesis are also discussed.

## Material and methods

### Tissue cultivation

Embryogenic suspension cultures were initiated from 28-day old calli in liquid Schenk and Hildebrandt (SH) medium,[6] supplemented with 30 × 10^−6^ mol/L Dicamba. They were treated with 0.085 and 0.17 mol/L NaCl according to Odjakova et al. [7] for 15 days until mature somatic embryos were formed. Cultures were incubated in darkness at 25 °C and 105 r/min on a shaker Gio Gyroty ® Shaker (New Brunswick Scientific).

### Protein isolation

Isolation of intracellular water soluble proteins was carried out on suspension cultures after 15 days of cultivation. Cells were grinded in liquid nitrogen and resuspended in 0.1 mol/L phosphate buffer, pH 8 [8] and centrifuged for 15 min at 15,000 × *g* at 4 °C to eliminate cellular residues. Supernatants containing diluted proteins were dialysed for 24 hours against a 1000-fold volume of phosphate buffer to eliminate low molecular weight thiol compounds.

### Diagonal electrophoresis

Diagonal polyacrylamide gel electrophoresis was carried out according to McDonagh [9] in two consecutive dimensions – non-reducing and reducing conditions and twice in non-reducing conditions for control.

### Estimation of protein thiols and disulphides

Initially, proteins were denatured with 6 M guanidine chloride in phosphate buffer to expose hidden cysteine residues. Free thiols were determined by mixing equal volumes of protein sample and 10 × 10^−3^ M 5,5′-dithiobis-(2-nitrobenzoic acid) (DTNB) in phosphate buffer (Ellman's reagent) and incubation for 15 min at room temperature, followed by absorbance measurement at 412 nm.[8] The concentration was calculated according to L-Cys standard curve. The total quantity of protein thiols was measured by the same protocol after preliminary reduction of protein and mixed disulphides with 20 × 10^−3^ M dithiothreitol (DTT) and dialysis for three hours. Concentration of thiols involved in disulphides was calculated as the difference between the total concentration and the concentration of free protein thiols.

### Bovine serum albumin glutathionylation

First, a bovine serum albumin (BSA) solution at 1 g L^−1^ concentration was treated with 20 × 10^−3^ M DTT for reduction of disulphide bonds for 30 min at room temperature. Following dialysis against phosphate buffer, pH 8 for 24 hours, oxidized (GSSG) or reduced glutathione (GSH) was added to a final concentration of 1 g L^−1^.[10] The reaction proceeded for 24 h at room temperature and was followed by dialysis. BSA glutathionylation was monitored in denaturing polyacrylamide gel electrophoresis.[11]

### Thioredoxin and glutaredoxin systems activity

The thioredoxin (Trx) and glutaredoxin (Grx) system activities were monitored spectrophotometrically, following the decrease in absorbance at 340 nm, caused by the oxidation of NADPH. The assay for Trx/Trx reductase system was prepared according to Arner et al. [12] using insulin as substrate. The assay for Grx was according to Zaffagnini et al.,[10] using glutathyonilated BSA as substrate. The decrease in absorbance was monitored for 1 min. All experiments were performed in triplicates. Amount of oxidized NADPH is calculated according to a previously prepared standard curve. For negative controls pre-reduced insulin for Trx/Trx reductase systems and non-glutathionylated BSA for Grx activity were used.

## Results and discussion

### Protein thiol disulphides formation

**Figure 1.**
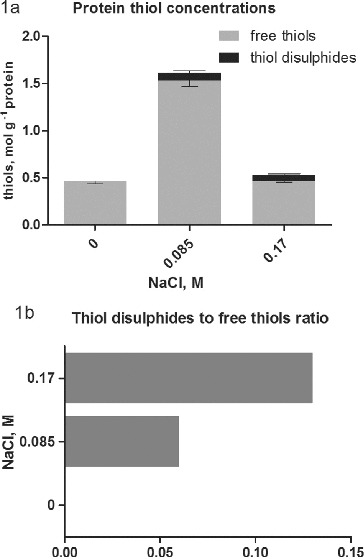
Concentrations of free protein thiols and thiol disulphides (either intramolecular disulphide bridges or thiols, engaged in mixed protein-glutathione disulphides (a) in salt-treated suspension cultures of *Dactylis glomerata* L. Columns represent mean values ±SD (*n* = 3). Thiol disulphides-to-free thiols ratio in proteins is represented in part (b).

The analysis of total thiols in intracellular proteins (Figure 1(a)) showed a sharp increase in the concentration to 1.6 mol g^−1^ protein in cultures treated with 0.085 mol/L NaCl compared to 0.4–0.5 mol g^−1^ in controls and cultures treated with 0 17 mol/L NaCl. This result can be explained by the appearance of a large amount of stress proteins at low salt concentration. Many of them, including thioredoxin and glutaredoxins,[13] and a number of other are Cys-rich proteins. The decrease of protein thiols concentration at 0.17 mol/L NaCl may be caused by the inhibition of protein synthesis at high salt concentrations [14] or to an exhaustion of the Cys pool for the synthesis of GSH. Increasing concentrations of GSH are observed in salt-treated plants including *Dactylis*.[15] In the last report it was also established that the concentration of free Cys is also elevated at this particular salt concentration, which may contribute to the reduced amount of protein thiols.

While in controls thiol disulphides were not observed, in salt-treated cultures such appeared (Figure 1(a)). Intracellular environment in general is highly reducing, which prevents the formation of disulphide bonds and such are not observed in water soluble proteins in the cytoplasm under normal conditions.[16] At least the concentration of protein thiol disulphides in controls is below the detection limit of the method used. In conditions of salt stress the concentration of ROS is expected to be increased.[2] This leads to oxidative changes in the sulphur-containing amino acids in the proteins.[17] The concentration of Cys, engaged in disulphides (Figure 1(a)), is even higher (0.08 mol g^−1^) in cultures, treated with 0.085 mol/L NaCl, compared to the higher salt concentration (0.06 mol g^−1^). However, the ratio of Cys disulphide to Cys is twice as low at 0.085 mol/L NaCl (Figure 1(b)). These results demonstrate the negative impact of high salt concentration on the physiological condition of the cultures in comparison with the low salt concentration, which has a favourable effect on somatic embryogenesis.[7] The method does not allow distinguishing of protein–protein disulphides from mixed ones. Glutathionylated proteins belong to the mixed disulphides, but also thiolated proteins could be expected. The high content of free Cys reported at high salt concentration [15] suggested possible protein thiolation. It is a relatively new direction in the research of oxidative changes.[18] It is especially important in plants and remains to be established. Overall, the current literature is giving more and more importance to the index of protein thiols.[16] It represents the molar ratio of low molecular weight thiols involved in mixed disulphides to proteins and free protein thiols. This value combined with the total intracellular redox potential[19] is considered as sensitive and important indicators of stress levels, cell fate and development phases.

**Figure 2.**
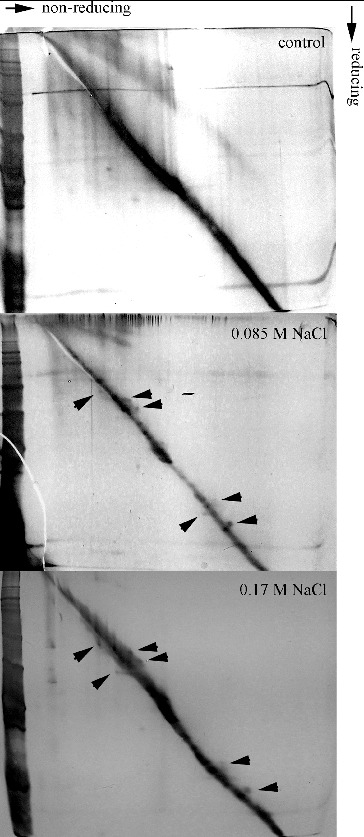
Diagonal gel electrophoresis for detection of protein thiol disulphides in salt-treated suspension cultures of *Dactylis glomerata* L. 10% T SDS PAGE were performed in two consecutive dimensions (non-reducing and reducing). Arrows indicates proteins above the diagonal (putative intramolecular disulphide bonds) and proteins below the diagonal (putative glutathionylated proteins). Gels were silver stained.

Additional analysis for the detection of protein thiol disulphides was performed by diagonal gel electrophoresis (Figure 2). The method is relatively new and depending upon the circumstances is applied to detect the kinetically stable proteins, and for analysis of intramolecular and intermolecular disulphide bridges, particularly in conditions of oxidative stress.[9] As expected protein with formed internal disulphide bridges should be located on the upper side of the diagonal, because a less compact conformation is acquired. Proteins, which were involved in intermolecular disulphide bridges or been glutathionylated, should be located on the underside of the diagonal after reduction.[9] In controls there were no proteins with altered electrophoretic mobility after reduction, whereas in cultures treated with 0.085 and 0.17 mol/L NaCl such occurred mainly above the diagonal and in a lesser extent below it (Figure 2).

These results showed that in the salt-treated cultures there is formation of intramolecular disulphide bonds. A similar effect was seen in many plants subjected to various types of stress as a result of oxidative signalling.[17,20] The formation of reversible disulphide bonds and glutathionylation are mechanisms for Cys residues protection from subsequent oxidation to sulphinic, sulphenic and sulphonic acids.[4] Such oxidation would have a negative and irreversible effect on protein functions. It should also be noted that the reversible formation of disulphide bonds is an important regulatory mechanism [21] and is probably related to stress tolerance and adaptation. As the pattern of proteins with altered electrophoretic mobility is similar in either salt-treated culture, this phenomenon should be attributed mainly to regulatory mechanism of particular proteins rather than occasional disulphide bond formation due to ROS production.

There are a rising number of reports, stating the importance of the intracellular redox potential in abiotic stress response [22] and somatic embryogenesis.[15] A potential link between oxidative signalling, protein glutathionylation and the formation of intramolecular disulphide bonds with development processes and especially with somatic embryogenesis in plants is yet to be explored, although it was implied by the authors cited.

### Thioredoxin and glutaredoxin systems activity

**Figure 3.**
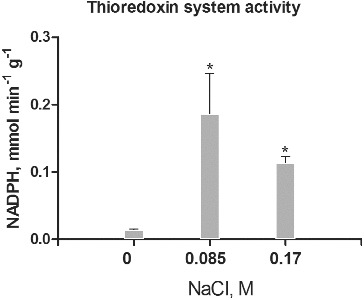
Thioredoxin/thioredoxin reductase system activity in salt-treated suspension cultures. Columns represent mean values and ±SD (*n* = 3). Significant values, compared to controls are indicated (*) as calculated by Student's *t*-test, *p* < 0.05. Activity is expressed as mmol oxidized NADPH per minute per g protein.

**Figure 4.**
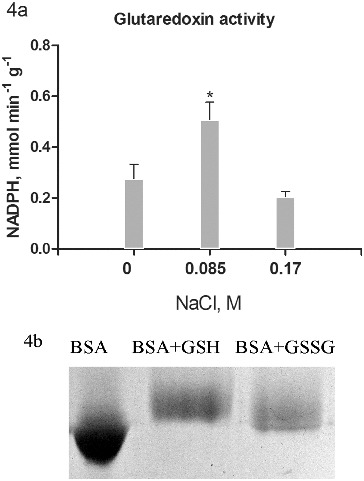
Glutaredoxin activity (a) in salt-treated suspension cultures. Columns represent mean values and ±SD (*n* = 3). Significant values, compared to controls are indicated (*) as calculated by Student's *t*-test, *p* < 0.05. Activity is expressed as mmol oxidized NADPH per minute per g protein. Glutathionylated bovine serum albumin was used as substrate. Glutathionylation of bovine serum albumin was monitored on 10% T SDS PAGE by the shift in the apparent molecular weight (b).

Thioredoxins and glutaredoxins represent Cys-containing proteins that provide reducing power for the reversible reduction of intramolecular disulphide bonds and protein-GSH mixed disulphides.[13] In this study the potency of the NADPH-dependent Trx/Trx reductase and Grx/GSH reductase systems in intracellular protein extracts from control and salt-treated cultures were measured (Figures 3 and  4). The data for the activity of Trx/Trx reductase showed that it is significantly higher in intracellular protein fractions of salt-treated cultures compared to controls (Figure 3). The low activity in controls correlated with the absence of protein Cys-disulphide (Figure 1(a)), but showed the constitutive expression of Trx in low concentrations and low activity, as was observed by other authors.[23] The higher activity in salt-treated cultures correlated with the response to salt stress. The relatively higher activity in cultures treated with 0.085 mol/L NaCl suggests adaptation to these stress levels as the ratio of thiol-to-thiol disulphides at this concentration is lower compared to cultures, treated with 0.17 mol/L NaCl (Figure 1(b)). The high Trx system activity was associated with response to different types of abiotic stress, as well as tolerance in different plant species.[23] Typically, a higher expression and activity of Trx were observed in salt-tolerant genotypes compared to salt-sensitive ones.[24] The higher activity in cultures treated with 0.085 mol/L NaCl in comparison to cultures treated with 0.17 mol/L NaCl also correlated with the higher accumulation of fresh mass, as well as the enhanced somatic embryogenesis.[15]

Additionally, Trx are involved in the regulation of several other proteins such as β-amylase,[25] which may also be related to the salt stress response and to the difference in the accumulation of fresh mass in salt-treated cultures of *Dactylis*.[15]

Potential substrates for the activity of the NADPH-dependent Grx/GSH reductase system are glutathionylated proteins which occur as a result of applied stress. Glutathionylation of pre-reduced BSA was conducted following the procedure of Zaffagnini [10] in a solution with GSSG and a parallel experiment with GSH. Effectiveness of glutathionylation is observed by electrophoresis, as it is expected that the glutathionylated albumin shows a lower electrophoretic mobility resulting from an increase in molecular mass by 305 Da for each GSH residue.[26] The results showed (Figure 4(b)) that GSH tends to give greater degree of glutathionylation.

Furthermore, BSA–GSH was used as substrate for Grx activity measurement (Figure 4(a)). The data for the activity of the Grx showed the highest values in cultures treated with 0.085 mol/L NaCl (Figure 4(a)). The increase was almost doubled compared to controls. Unlike the Trx system, cultures treated with 0.17 mol/L NaCl showed even lower activity compared to controls. There was a relatively high activity observed in all treatments, confirming the importance of reversible protein S-glutathionylation in many regulatory processes of growth and development.[27] Glutathionylation of certain proteins probably relates to the process of somatic embryogenesis as well.[28] Overall, the increased activity of Grx in cultures treated with 0.085 mol/L NaCl was similar to the Trx activity increase and had the same importance in response to salt stress.[29] The high activity of Trx prevents protein oxidative damage under conditions of stress and contributes to higher tolerance.[4] Experiments, related to transgenic expression of Grx genes from one species to another had also shown the importance of these proteins for enhanced stress tolerance.[30]

## Conclusions

In summary, our results show that salt stress leads to a significant change in protein thiol status of water soluble intracellular proteins in suspension cultures of *Dactylis glomerata*. Treatment with either low (0.085 mol/L) or high (0.17 mol/L) NaCl concentrations resulted in increased protein thiols content and disulphide bond formation. Accordingly, the activity of the thioredoxin and glutaredoxin systems was increased in salt-treated cultures, with the highest activity at 0.085 mol/L NaCl. Besides the significance for stress response and adaptation, these changes may be also related to the increased formation of somatic embryos at 0.085 mol/L NaCl.
